# Preparing for the SARS-CoV-2 pandemic: creation and implementation of new recommendations

**DOI:** 10.1007/s00540-020-02827-2

**Published:** 2020-07-20

**Authors:** David Conrad, Patrick Hoffmann, Ulrich Berwanger, Tobias Hüppe, Thomas Volk, Tobias Fink

**Affiliations:** Department of Anesthesiology, Intensive Care and Pain Therapy, Saarland University Medical Center and Saarland University Faculty of Medicine, Kirrberger Strasse 1, 66421 Homburg/Saar, Germany

**Keywords:** Anesthesia, COVID-19, SARS-CoV-2, Pandemic, Intubation

## Abstract

During the SARS-CoV-2 pandemic in 2020, departments of anesthesiology worldwide have encountered new and unique challenges. In this short communication, we present and assess our recommendations for orotracheal intubation, a frequent high-risk procedure. We will point out that interdisciplinary cooperation with “non-patient care” departments like the Institute for Medical Microbiology and Hygiene tremendously helped us in creating this and other new, clear standards for anesthesiological procedures. Moreover, to reliably implement our newly created measures, we distributed incisive posters and organized comprehensive training sessions. Eventually, we summarize and analyze the occurring problems of our suggestions for intubation during their realization.

With expertise in airway management, intensive care, emergency, and respiratory medicine, anesthesiologists are essential for coping with challenges like the recent COVID-19 pandemic. However, because of the airborne transmission of COVID-19, anesthesiologists are at a high risk of getting infected [1–3]. Moreover, the COVID-19 disease was a new and unexpected threat, and applicable guidelines were not available at the beginning of the outbreak [4–7].

To protect staff especially in anesthesia, e.g., during high-risk procedures like the orotracheal intubation [3], a lot of new standard operating procedures (SOP) were defined.

As an example, we would like to present the development and implementation of our recommendations for the “intubation of COVID-19 patients—suspected or confirmed” (Fig. 1).

**Fig. 1:**
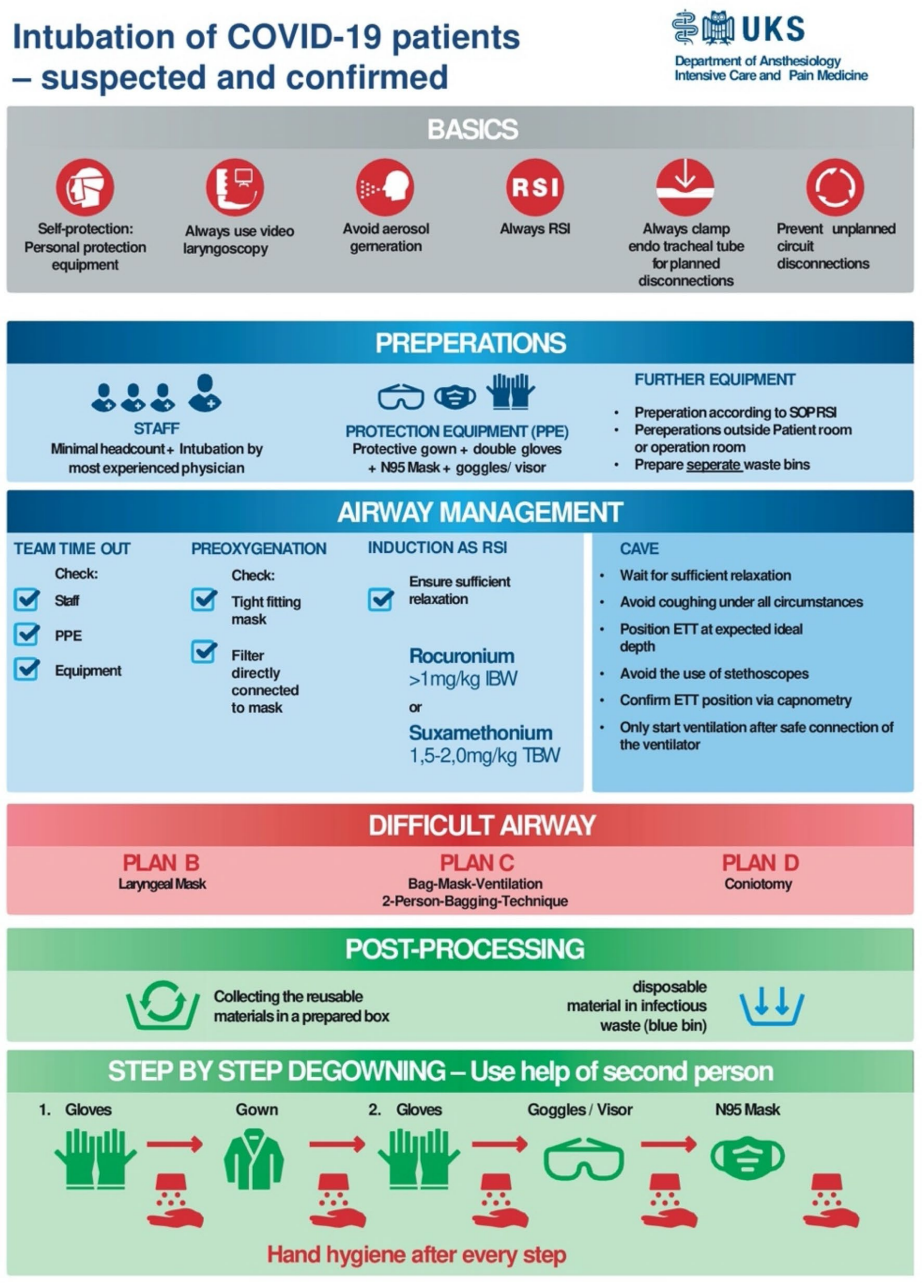
Intubation. The figure describes the procedure of intubation of patients with COVID-19. The principles are placed right at the beginning (gray). Most importantly, they include self-protection and the use of video laryngoscopy. The preparations follow. These concern staff, PPE, and the preparations. Before the induction of anaesthesia, a team time out should be set up. The actual induction of anaesthesia is performed as “Rapid Sequence Induction”. It is particularly important to avoid aerosol formation and the resulting contamination of the environment (blue). If intubation is difficult, insertion of a supraglottic airway device is the first choice (plan B) (red). Subsequently, the correct disposal of the consumables and the correct removal of personal protective clothing are important too (green)

Our focus was not to create an entirely new procedure in the first place, but rather adapt an existing SOPs to the new and unique circumstances, following available international recommendations [8, 9]. At this point, cooperation between our specialist discipline and the Department of Medical Microbiology and Hygiene as a “non-patient care” department with high expertise in hygienic matters proved to be extraordinarily valuable. We accomplished this successful cooperation by sending one of our consultants there, to work with hygienic staff deliberately on the evaluation and optimization of our new standards. This consultant worked there for 1 month. Thus, both problems with practical implementation and adherence to hygienic recommendations could be re-evaluated and adapted quickly, sometimes within hours. For example, specialists of the department of hygiene were not aware of the logistics and operational processes in the operation theaters, while anesthetists, who in Germany are also intensive care and emergency physicians, have broad interdisciplinary knowledge of the logistics and proceedings in different areas of the hospital. Hence, this cooperation resulted in useful and practice-based recommendations. Not only the ones presented here (Figs. 1, 2), but also in a variety of recommendations, such as hygiene measures in the different areas (e.g., holding area and recovery room).

**Fig. 2:**
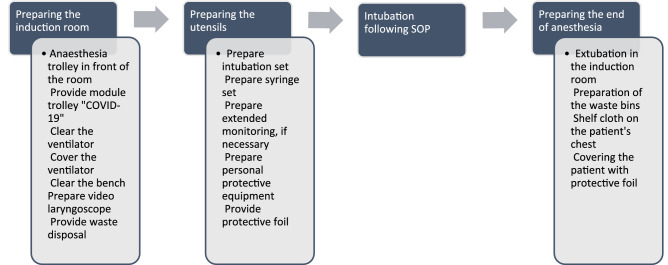
Scheme: perioperative procedure. The figure shows the procedure from the preparation and provision of all materials to the induction of anaesthesia and the end of anaesthesia

To ensure proper implementation in all areas, our recommendations were presented as large posters within all areas where orotracheal intubation is expected (e.g., operation theatres, intensive-care unit, and emergency room). We focused on an easy to understand layout, showing all critical points concerning preparation, execution, and post-processing.

First and foremost, we focused on self-protection and avoiding droplets. The six basic measures to provide these are displayed right at the head of the poster (Fig. 1, grey color). The blue-colored part shows the preparation and execution of actual airway management. Especially proper checks and team timeouts are vital here, even (probably particularly) in time-critical emergency situations. During these time outs, all preparatory steps, personal protective equipment (PPE), and necessary equipment have to be addressed and confirmed. Additionally, core issues like the avoidance of coughing, confirming tube placement with capnography, etc. (see dark blue section) should be reconsidered.

In airway management, there must be backup plans if the initial intubation attempt fails (Fig. 1, red color). We regard the avoidance of droplet, and aerosol emergence as critical, which is why inserting a laryngeal mask (PLAN B) right away is considered more appropriate than intermittent bag-mask ventilation (PLAN C).

Post-processing and proper de-gowning are at least as important as wearing the PPE in the first place. Therefore, we think that it is essential to illustrate it separately (Fig. 1, green section). These recommendations came directly from the Department of Hygiene. While several sources, especially in social media, suggested to continuously disinfect the gloves until every part of the PPE is taken off; we learned from our colleagues that using disinfectant more than once will lead to damage and subsequently to impairment of protection of the nitrile gloves.

To provide a holistic approach and to complement the recommendations for intubation, further preparations were made for the perioperative handling of COVID-19 patients (Fig. 2). A new modular trolley was created. Here separately prepared and shrink-wrapped sets are kept in stock. Accordingly, we are able to follow the procedure step-by-step and package-by-package, which further enhanced safety. Moreover, cabinets and anesthesia trolleys within the induction rooms did not have to be used. Thus, we were able to prevent contamination of our equipment in the induction rooms. The specialists in the department of hygiene explained how droplets in the induction rooms could lead to contamination and suggested the now used package-by-package-approach. Furthermore, we prevented contamination of the operation room using two teams of anesthetists. One team only performed induction of anesthesia and the extubation of the patient, both in the induction room, while the other team maintained the anesthesia during the surgery.

The following sets are kept in the trolley:

- Personal protective equipment (PPE) set.
- Preoxygenation set.
- Intubation set.
- Peripheral venous access kit.
- Sets for invasive blood pressure measurement, central venous lines, or urinary catheters.
- Syringe sets with drugs for the induction, including emergency drugs and standardized syringe labels.

To implement new procedures in everyday clinical practice, simulation training is of main importance [10]. The unusual handling of personal protective equipment and the new procedures for the induction of anesthesia were, therefore, trained using in situ simulation. For over 1 week, almost all consultants, interns, and nurse-staff were trained in setup training scenarios. Besides, the safe donning and doffing of protective clothing was made available in text and image form throughout the clinic.

Creating new recommendations and standards may be a significant part of overcoming unusual circumstances complicating our everyday work as anesthesiologists. However, once created, they must be implemented and fit in daily routine. To address these challenges, we conclude that the concept of performing extensive simulation trainings was very beneficial. Furthermore, the success of implementing new concepts and recommendations mainly depends on the acceptance of and consent with the staff [11], as the close cooperation with our the Institute of Microbiology and Hygiene was essential. It enabled us to handle staff feedback, where medical recommendations concerning hygiene and patient treatment did not match with local preconditions. Adjusting our recommendations to the pre-existing circumstances in combination with our in situ simulation training resulted in outstanding acceptance and excellent transfer of the new recommendations into the everyday routine.

However, all the measures mentioned above require a high level of staffing and time. To date, our novel standardized procedures completely prevented coronavirus transmission during high-risk procedures (*n* = 0).
